# The tumor-modulatory effects of Caspase-2 and Pidd1 do not require the scaffold protein Raidd

**DOI:** 10.1038/cdd.2015.31

**Published:** 2015-04-10

**Authors:** L Peintner, L Dorstyn, S Kumar, T Aneichyk, A Villunger, C Manzl

**Affiliations:** 1 Division of Developmental Immunology, Medical University of Innsbruck, 6020 Innsbruck, Austria; 2 Centre for Cancer Biology — An Alliance between SA Pathology and the University of South Australia, Adelaide, SA 5001, Australia; 3 Division of Molecular Pathophysiology, Biocenter, Medical University of Innsbruck, Innsbruck 6020, Austria; 4 Department of General Pathology, Medical University of Innsbruck, Innsbruck 6020, Austria

## Abstract

The receptor-interacting protein-associated ICH-1/CED-3 homologous protein with a death domain (RAIDD/CRADD) functions as a dual adaptor and is a constituent of different multi-protein complexes implicated in the regulation of inflammation and cell death. Within the PIDDosome complex, RAIDD connects the cell death-related protease, Caspase-2, with the p53-induced protein with a death domain 1 (PIDD1). As such, RAIDD has been implicated in DNA-damage-induced apoptosis as well as in tumorigenesis. As loss of *Caspase-2* leads to an acceleration of tumor onset in the *Eμ-Myc* mouse lymphoma model, whereas loss of *Pidd1* actually delays onset of this disease, we set out to interrogate the role of *Raidd* in cancer in more detail. Our data obtained analyzing *Eμ-Myc/Raidd*^−/−^ mice indicate that *Raidd* is unable to protect from c-Myc-driven lymphomagenesis. Similarly, we failed to observe a modulatory effect of Raidd deficiency on DNA-damage-driven cancer. The role of Caspase-2 as a tumor suppressor and that of Pidd1 as a tumor promoter can therefore be uncoupled from their ability to interact with the Raidd scaffold, pointing toward the existence of alternative signaling modules engaging these two proteins in this context.

A number of mechanisms have evolved to trace and remove potentially dangerous cells. Deregulation of the induction of apoptosis upon oncogenic stress, for example, can facilitate the accumulation of cells prone to undergo malignant transformation. Cell death by apoptosis depends on the cascade-like activation of proteases of the Caspase family.^1^ Among these, the evolutionarily most conserved protease, Caspase-2, turns out to be a potent tumor suppressor in mice^2, 3, 4, 5, 6, 7^ and correlative expression data support a conserved role in human cancer.^8, 9, 10, 11, 12, 13^

Early studies suggested that Caspase-2 interacts with other proteins for its activation (e.g., after genotoxic stress), but the protease seems also able to auto-activate cell death on its own when present in sufficiently high concentration.^14, 15, 16, 17, 18^ The most prominent Caspase-2-containing protein complex was dubbed the ‘PIDDosome' and described to contain the p53-induced protein with a death domain (PIDD1) and receptor-interacting protein-associated ICH-1/CED-3 homologous protein with a death domain (RAIDD, also known as CRADD).^19^ Although the molecular details of the pro-apoptotic potential of Caspase-2 are still discussed and alternative roles in the DNA-damage response, cell cycle arrest or sensor of metabolic stress are mechanistically poorly understood, Caspase-2 clearly limits tumorigenesis in different settings. These include aberrant expression of c-Myc in B cells^3, 4^ or deletion of the DNA-damage response regulator, ataxia telangiectasia mutated kinase (ATM), both driving lymphomagenesis^6^ as well as overexpression of the Her2/ErbB2 oncogene in breast^5^ or that of mutated KRAS in the lung epithelium, driving carcinoma formation.^7^ One of these studies, addressing also the role of Pidd1 in c-Myc-driven lymphomagenesis, revealed an unexpected oncogenic role for Pidd1, thereby questioning the physiological relevance of the PIDDosome complex in Caspase-2-mediated cell death and tumor suppression.^4, 20^ However, the exact role of the scaffold protein Raidd within these processes remains unaddressed so far.

Raidd, a bipartite adapter containing a death domain (DD) and a caspase-recruitment domain (CARD) was first described to bind to the DD-containing kinase RIPK1 and the *C. elegans* caspase CED-3,^21^ supporting a role in cell death initiation. Subsequently, the interaction of Caspase-2 and Raidd was biochemically proven^22^ and proposed to be required for Caspase-2 autoprocessing preceding its activation.^19^ More recent studies propose an anti-inflammatory role for Raidd through suppression of nuclear factor kappa-light-chain enhancer (NF-κB) activation and cytokine production upon T-cell receptor stimulation by negatively interfering with the Carma1/Malt1/Bcl-10 signaling complex.^23, 24^

First evidence for a potential role of RAIDD in human cancer was discovered in a biochemical screen using mantle cell lymphomas, which detected a downregulation of RAIDD by microarray analysis,^10^ whereas others reported on RAIDD-linked multidrug resistance in osteosarcoma cells.^25^ Furthermore, tumor cell apoptosis induced by inhibitors of histone de-acetylases in treatment-resistant adult T-cell leukemia lines reportedly required Caspase-2 and Raidd.^26^ It is also reported that the Caspase-2/Raidd axis is necessary after ER stress, for example, in the course of infection with the oncolytic maraba virus.^27^

Taken together, these studies support a role for RAIDD in drug-induced cancer cell death as well as in tumor suppression, most likely linked to its role as a direct activator of Caspase-2. Alternatively, RAIDD may negatively interfere with PIDD- or BCL10-regulated NF-κB signaling^23, 24, 28^ and thereby suppress pro-tumorigenic inflammation. To address the role of Raidd in tumorigenesis in more detail, we exploited different mouse models where we induced thymic lymphomas by *γ*-irradiation, fibrosarcomas by 3-methylcholanthrene (3-MC) injection or B-cell lymphomas by aberrant expression of the c-Myc proto-oncogene. Our results suggest that Raidd is not a suppressor of tumors in the mouse models tested.

## Results

### Loss of Raidd has no impact on tumor formation after DNA damage

**Figure 1:**
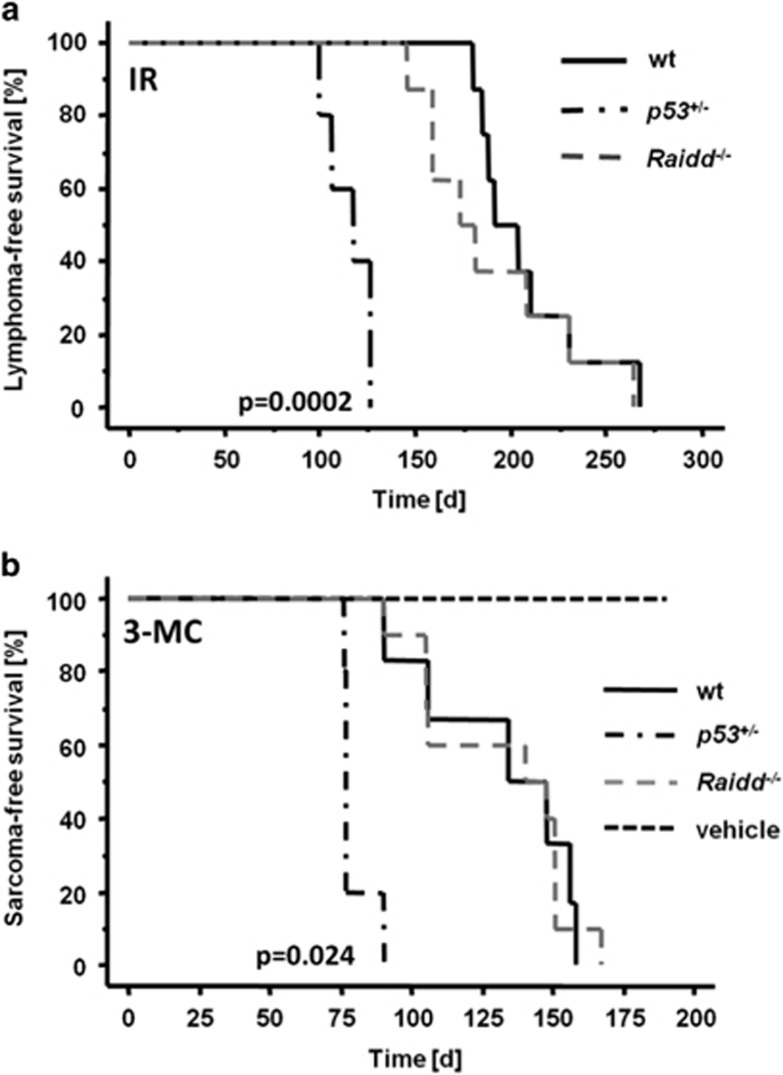
Loss of Raidd does not impact on DNA-damage-induced tumor formation. **(a)** Kaplan–Meier plot of tumor-free survival in response to repeated low-dose *γ*−irradiation (4 × 1.75 Gy) of wt (*n*=8, mean=207 days), *Raidd*^−*/*−^ (*n*=8, mean=190 days) and *p53*^*+/*−^ mice (*n*=5, mean=115 days). **(b)** Kaplan–Meier analysis of 3-methylcholanthrene (3-MC)-treated mice. Wild type (*n*=6, median=132 days); *Raidd*^−*/*−^ (*n*=10, mean=131 days); *p53*^*+/*−^ (*n*=5, mean=79 days). Wild-type mice injected with vehicle only (*n*=3) were used as solvent controls

As Raidd was reported to have a role in DNA-damage-induced apoptosis because of its ability to form a complex with Pidd1 and Caspase-2,^19^ we used different cancer models to evaluate the impact of Raidd in tumorigenesis induced by DNA lesions. Induction of thymic lymphomas was triggered in 4-week-old mice by repeated low-dose irradiation (1.75 Gy). As reported before, tumor formation was significantly accelerated in *p53*^*+/*−^ mice when compared with wild-type (wt) controls, showing a mean survival of 115 and 207 days, respectively. Absence of *Raidd*, however, had no effect on the onset of tumorigenesis (Figure 1a) or tumor immunophenotype (data not shown) and animals developed thymic lymphomas with a mean survival of 190 days, similar to their wt littermates (*P*=0.37).

In another tumor model, we treated adult mice with a single injection of 3-MC causing the formation of bulky adducts in DNA leading to fibrosarcoma formation within 6 months. As reported before,^29^ *p53*^*+/*−^ mice developed 3-MC-induced sarcomas significantly earlier but loss of Raidd failed to accelerate tumor onset when compared with wt controls (Figure 1b; *P*=0.97).

Together with our previously published data^4^ this demonstrates that the PIDDosome, and each of its components individually, are unable to prevent DNA-damage-induced tumor formation inflicted by *γ*-irradiation or bulky DNA-adduct formation.

### c-Myc-induced lymphomagenesis is limited by Caspase-2 in a Raidd-independent manner

Caspase-2 and Pidd1 were reported to modulate lymphomagenesis in *Eμ-Myc* mice, a model somewhat mimicking human Burkitt lymphoma.^4^ Hence, we introduced Raidd deficiency into *Eμ-Myc* transgenic animals to explore its impact on tumorigenesis caused by oncogenic stress. Excess proliferation by aberrant c-Myc expression in premalignant mice is usually counterbalanced by increased cell death rates providing a rational why loss of pro-apoptotic proteins, for example, of the BH3-only group, accelerates disease onset.^30, 31, 32, 33^

**Figure 2:**
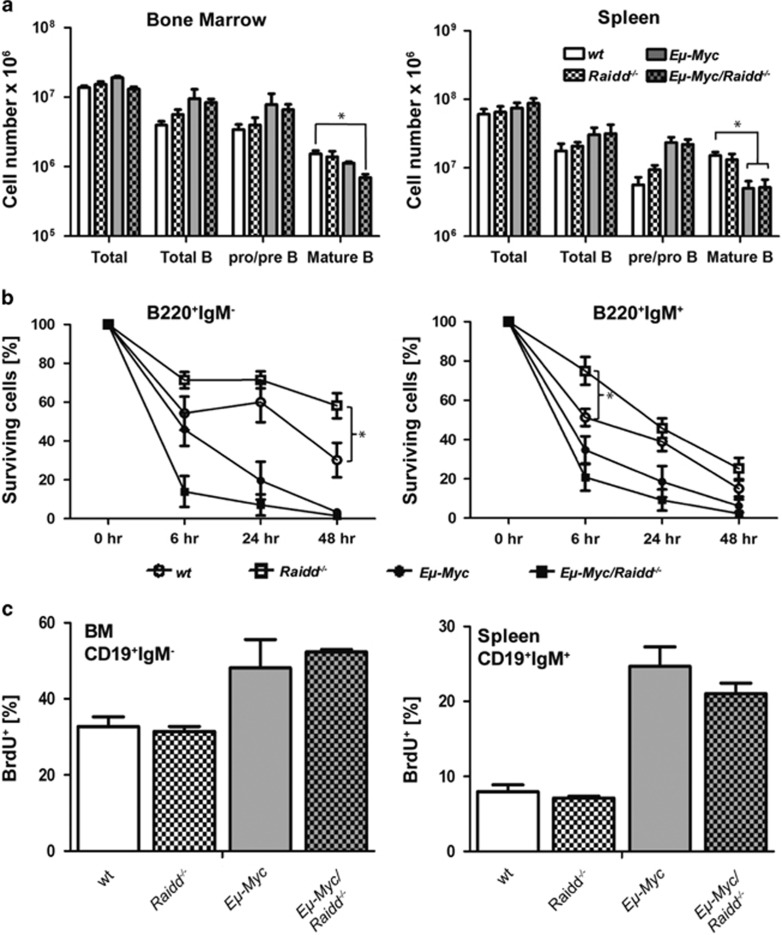
B-cell subset composition, proliferation and cell death responsiveness in premalignant *Raidd*^−*/*−^ mice. **(a)** The development and the distribution of B cells in bone marrow and spleen was assessed in 4-week-old *Eμ-Myc* transgenic mice lacking or expressing Raidd. Single-cell suspensions of the organs were counted and stained with cell surface marker-specific antibodies and analyzed by flow cytometry. Bars represent means (*n*=3-5 per genotype)±S.E.M. **(b)** Sorted premalignant B cells (pre-B from bone marrow, CD19^+^IgM^−^CD43^−^ or immature B cells CD19^+^IgM^low^ from spleen) were analyzed for spontaneous death after 6, 24 and 48 h using Annexin-V plus 7-AAD staining in a flow cytometer. Symbols represent means of *n*=3-5 per genotype±S.E.M. **(c)** Proliferation rates were quantified 4 h after a single injection of BrdU in bone-marrow-derived pro/pre-B cells, (CD19^+^IgM^−^) or (CD19^+^IgM^+^) B cells from spleen. Bars represent means (*n*=3-5) per genotype±S.E.M.

To evaluate the role of Raidd early in disease development, the B-cell subset composition was assessed in premalignant *Eμ-Myc/Raidd*^−*/*−^ mice. In line with published data,^4, 32, 34, 35^ the numbers of B220^+^IgM^−^ pro/pre-B cells were increased in bone marrow and spleen, whereas numbers of mature B cells were diminished in the periphery of *Eμ-Myc* mice when compared with wt controls (Figure 2a). However, no other gross alterations within the B-cell composition and distribution in *Eμ-Myc* mice specifically lacking Raidd were noted.

To monitor B-cell survival upon oncogenic stress in the absence or presence of Raidd, we next sorted pre-B and immature B cells of premalignant mice and put them in culture. Notably, B cells from non-transgenic *Raidd*^−*/*−^ mice appeared to be slightly less sensitive to spontaneous cell death when compared with wt (Figure 2b). As reported before,^30, 31, 32^ B cells derived from *Eμ-Myc* mice died more rapidly when compared with the non-transgenic counterparts but additional lack of Raidd had no relevant impact (Figure 2b). Similarly, Raidd deficiency had no impact on homeostatic or c-Myc-driven B-cell proliferation, as assessed by BrdU-incorporation analysis in immature CD19^+^IgM^−^ and CD19^+^IgM^+^ B cells from bone marrow or spleen of premalignant mice (Figure 2c).

**Figure 3:**
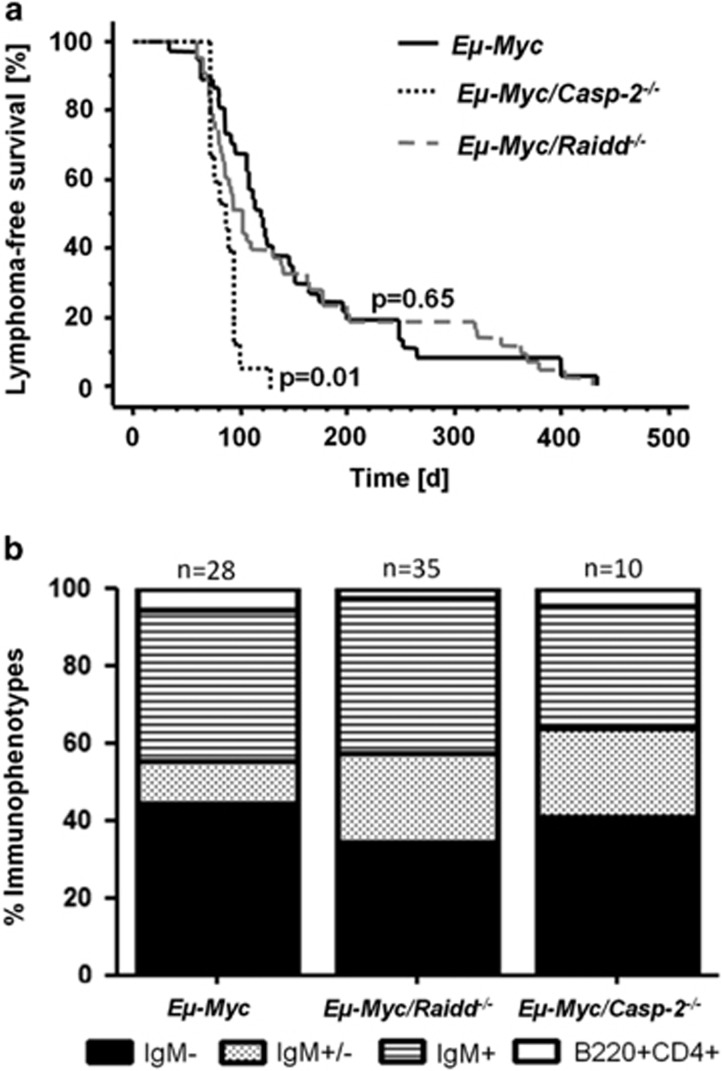
Suppression of c-Myc-induced B-cell lymphoma formation by Caspase-2 does not depend on Raidd. **(a)** Lymphoma-free survival of *Eμ-Myc* (*n*=37, mean=128 days), *Eμ-Myc/Raidd*^−*/*−^ (*n*=43, mean=124 days) and *Eμ-Myc/Casp-2*^−*/*−^ mice (*n*=15, mean=101 days). **(b)** Flow cytometric analysis of tumor immunophenotype. A comparison of the frequency of the different immunophenotype across genotypes by Chi-square test revealed differences between wt and *Casp-2*^−*/*−^ tumors (*P*=0.043), whereas the overall distribution between wt and *Raidd*^−*/*−^ tumors was not significantly different (*P*=0.1)

Next, cohorts of *Eμ-Myc*, *Eμ-Myc/Raidd*^−*/*−^ and, for reference, *Eμ-Myc/Casp-2*^−*/*−^ mice were monitored for tumor onset. Strikingly, although loss of Caspase-2 leads to accelerated disease onset (mean survival: 101 d), loss of Raidd had no effect, when compared with Raidd-proficient *Eμ-Myc* mice presenting with a mean survival: 128 *versus* 124 days (Figure 3a). White blood cell counts and tumor burden were also comparable between genotypes (Supplementary Figures 1a and b).

Tumors were subsequently classified by flow cytometry as either pre-B cell (IgM^−^), immature B cell (IgM^+^), mixed (IgM^+/−^) or as early hematopoietic progenitor-derived lymphomas (B220^+^CD4^+^). *χ*^2^ analysis confirmed differential distribution between wt and Casp-2^−/−^ tumors (*P*=0.043), as reported before^4^ but no significant difference was noted between wt and Raidd^−/−^ tumors (Figure 3b).

**Figure 4:**
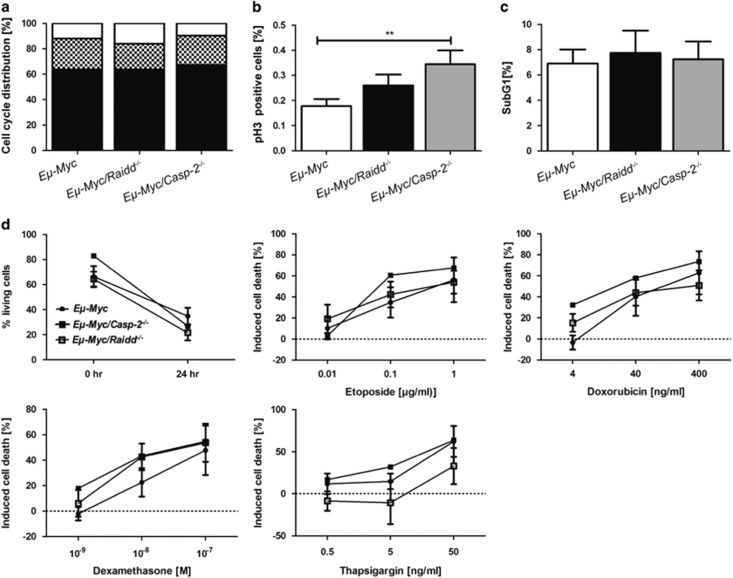
Normal cell cycle distribution and tumor cell apoptosis in *Eμ-Myc* tumor cells lacking Raidd. **(a)** Cell cycle phases G1 (black), S (dotted) and G2/M (white) of lymphoma cells were assessed *ex vivo* by intracellular DNA content analysis in tumors derived from *Eμ-Myc* (*n*=21), *Eμ-Myc/Raidd*^−*/*−^ (*n*=22), *Eμ-Myc/Casp-2*^−*/*−^ (*n*=10) mice. **(b)** To define the percentage of cells in M-phase, *ex vivo* tumor cells were stained with an antibody specific for the phosphorylated variant of Histone H3 (pH3) and propidium iodide. Bars represent mean values of pH3^+^ cells±S.E.M. *Eμ-Myc* (*n*=17), *Eμ-Myc/Raidd*^−*/*−^ (*n*=11) *Eμ-Myc/Casp-2*^−*/*−^ (*n*=5). **(c)** Apoptosis *in situ* was assessed by Sub-G1 analysis of freshly isolated lymph node tumor masses followed flow cytometric analysis (wt *n*=24, *Raidd*^−*/*−^ *n*=22, *Casp-2*^−*/*−^ *n*=10) mean values±S.E.M. **(d)** Freshly isolated lymphoma cells were cultivated on feeder cells for 24 h and were either left untreated (upper left graph) or exposed to increasing doses of the indicated chemo-therapeutics. Cell death was assessed by Annexin-V/7-AAD staining combined with anti-CD19 to identify B cells. Symbols represent mean values, *n*>3–7 for each genotype±S.E.M.

Further, in lymphoma cells derived from *Eμ-Myc/Raidd*^−*/*−^ mice, cell cycle distribution (Figures 4a and b), spontaneous (Figure 4c) and drug-induced cell death (Figure 4d) were monitored and found comparable to their wt counterparts. Notably, Caspase-2-deficient tumors showed again an increase in phospho-Histone H3 staining, suggesting perturbed cell cycle control (Figure 4b). In line with a lack of impact on tumor latency, loss of Raidd did not release the pressure to inactivate p53, a known secondary hit, usually observed in about 25% of all *Eμ-Myc* lymphomas.^36^ Inactivation of p53, as assessed by western blotting for p53 and p19ARF, was seen at similar rates in *Eμ-Myc* (26%, *n*=11) and *Eμ-Myc/Raidd*^−*/*−^ (27%, *n*=19) tumors (Supplementary Figures 1c and d).

Overall, these data demonstrate that the adaptor protein Raidd is not limiting Myc-driven tumorigenesis thereby uncoupling the tumor-suppressor function of Caspase-2 from Raidd-dependent autoactivation and the oncogenic potential of Pidd1 from Raidd-modulated NF-*κ*B signaling.

### Loss of Caspase-2 promotes aneuploidy

As Caspase-2 has been implicated in mediating mitotic catastrophe as a response to DNA-damage after failed cell cycle arrest^37, 38^ and that c-Myc overexpression drives proliferation stress leading to DNA damage,^39^ we investigated if lack of Caspase-2 would influence genomic stability of normal, premalignant or transformed cells.

**Figure 5:**
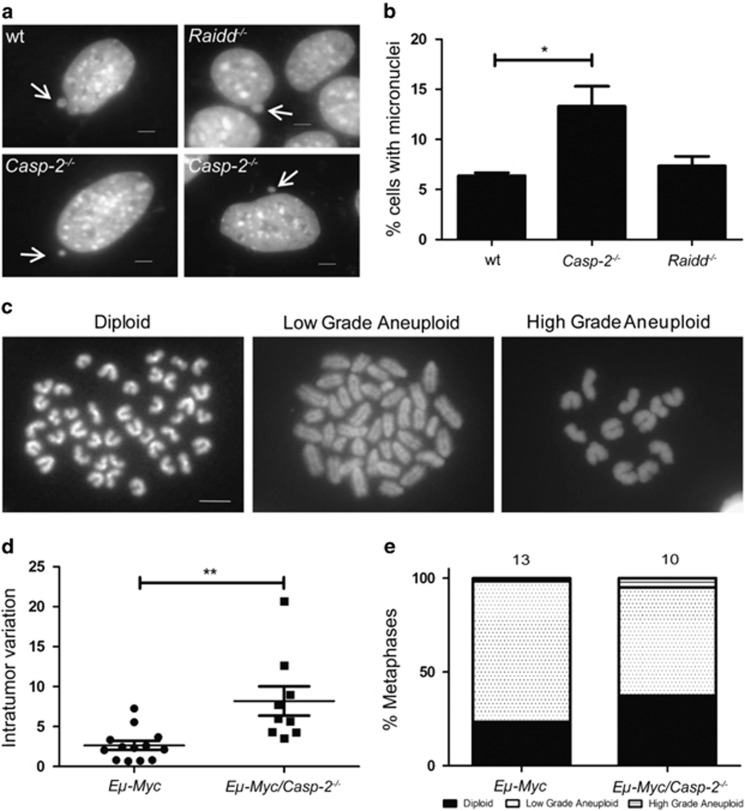
Caspase-2-loss leads to increased micronuclei formation and aneuploidy. (**a**) Representative images of micronuclei formation in SV40 MEFs of the indicated genotypes stained with 4′,6′-diamidino-phenylinodole. Arrows indicate micronuclei formation in wt MEFs. Scale bars=5 *μ*m. (**b**) Quantification of data assessed in **a**. A minimum of 300 cells per genotype was evaluated. Wt *versus Casp-2*^−*/*−^ (*P*=0.03; Student's *t*-test). Bars represent mean of *n*>4–6 for each genotype±S.E.M. (**c**) Representative images of chromosome spreads using freshly isolated *Eμ-Myc* lymphoma cells. Metaphase spreads are showing representative euploid (left panel) or aneuploidy karyogramms ranging from low (middle panel) to high-grade (right panel). Scale bar=5 *μ*m. A minimum of 50 spreads per genotype was evaluated. (**d**) Variation of counted chromosome numbers within single tumors from *Eμ-Myc* (*n*=13) *versus Eμ-Myc/Casp-2*^−*/*−^ (*n*=10) *mice* (*P*=0.0029; Student's *t*-test). (**e**) Quantification of the variance of chromosome numbers in tumors derived from indicated genotypes. A *χ*^2^ test showed a significant difference between wt and *Casp-2*^−*/*−^ (*P*<0.0001)

First, we investigated chromosomal stability in SV40-immortalized mouse embryonic fibroblasts (MEFs) from wt and Caspase-2-deficient mice (Figures 5a and b). Numbers of micronuclei, which emerge after errors during mitosis and result in individual chromosomes or fragments outside of the main nucleus, were significantly increased in cells lacking Caspase-2 in comparison to wt cells. *Raidd*^−*/*−^ MEFs did not show increased susceptibility to micronuclei formation (Figure 5b). Chromosomal stability was further examined by counting chromosome numbers in metaphase spreads in B-cell lymphomas (Figures 5c–e). *Eμ-Myc* transgenic B-cell tumors deficient for Caspase-2 did show a higher variation of chromosome numbers within individual tumor samples. Taken together, these data are consistent with published results, indicating that Caspase-2 acts in maintaining genomic stability.^37^

In an attempt to identify potential effectors of Caspase-2 in this process and based on the reported role of Caspase-2 in p53 activation and target gene expression upon DNA damage,^40^ we performed qPCR analysis as well as unbiased genome-wide expression analysis comparing mRNA from premalignant splenic IgM^+^D^−^ B cells of *Eμ-Myc* transgenic animals deficient or proficient for Caspase-2 or Raidd. We started by comparing expression levels of mRNAs of *p21, Noxa* and *Puma* by qRT-PCR, anticipating that c-Myc-driven p53 activation would result in reduced expression of these targets in the absence of Caspase-2.^40^ This, however, was not the case, or at least the noted differences did not reach statistical significant differences (Supplementary Figure 2). Comparison of gene-chip data revealed only few candidates, including *Fbxw10, Fgd6, Hist3h2a, Hmha1, Igf1R, Lyrm7, RhoBTB1, Slc25a13* and *Zfp39*, that were deregulated more than twofold in the absence of Caspase-2 or RAIDD when compared with wt (Supplementary Figure 2). Only one of these candidates, that is, RhoBTB1, was subsequently confirmed by qPCR analysis. However, as RhoBTB1 is unlikely related to genomic stability this was not followed up in detail during the course of this study. This indicates that there are no significant changes, at least in the premalignant B cells analyzed that may contribute to the genomic instability observed following loss of Caspase-2.

## Discussion

In this study, we investigated the relevance of Raidd in tumorigenesis sparked by its function as an adaptor protein for Caspase-2 and Pidd1, both being reported to influence tumorigenesis *in vivo*. Caspase-2 is well characterized as a tumor-suppressor gene in various mouse cancer models,^3, 4, 5, 6, 7^ whereas loss of Pidd1 leads to a delayed tumor onset upon c-Myc overexpression.^4^ Our data demonstrate that loss of Raidd has no influence on the development of radiation-induced thymic lymphomas or chemically induced fibrosarcomas (Figure 1). Although the mean latency in the absence of Raidd appeared to be shortened when compared with wt (190 *versus* 205 days), statistical significance was not reached, possibly due to the rather small cohort size, requiring more detailed follow up. Yet, these observations are similar to the published results in Caspase-2- and Pidd1-deficient mice^4^ and exclude a role of any of the PIDDosome components in these DNA-damage-driven models of cancer.

Strikingly, Raidd deficiency also had no influence on tumor latency in c-Myc-driven B-cell lymphomas (Figure 3a), contrasting the findings with Caspase-2- or Pidd1-deficient mice, where Caspase-2 acts as a tumor suppressor and Pidd1 as a tumor promoter in this model. Our data show that loss of Raidd is dispensable for the development of B-cell lymphomas in the *Eμ-Myc* mouse model and that Raidd deficiency has no impact on the development, proliferation and cell death of premalignant and transformed cells (Figure 2). The results thus indicate that Caspase-2-mediated tumor suppression is independent of both Pidd1 and Raidd. This is in line with the idea that Caspase-2 autoactivation can occur without the need for additional accessory proteins.^14, 15, 16^ Consistent with our conclusion, it has been shown that Caspase-2 indeed appears to be able to exert biological effects that do not depend on either catalytic activity or interaction with RAIDD or PIDD1, exemplified by translational control of p21 levels.^41^ As reduced levels of p21 by Caspase-2 knockdown sensitizes Hct116 colon cancer cells to irradiation-induced cell death,^41^ one may speculate that a similar effect may account for the tumor-suppressive effect noted in *Eμ-Myc/Casp-2*^−*/*−^ mice. However, deficiency in *Cdkn1a*, encoding for p21, has no impact on the tumor latency in *Eμ-Myc* mice,^42^ excluding this possibility. However, it remains possible that not all aspects of Caspase-2 biology are conserved between mice and humans, as previously also recognized for the Caspase-2-dependent Chk1-suppressed cell death pathway that appears functional in human cancer cells but not in mouse lymphocytes or MEF.^43^

It is also not easy to reconcile the findings connecting deregulated expression of RAIDD in human cancer development and drug-resistant phenotypes^10, 25, 26, 27^ with our data presented here. Although deregulated expression of RAIDD levels in cancer may be a bystander effect, for example, due to de-differentiation of cancer cells or deregulation of multiple signaling pathways ultimately also impacting on RAIDD mRNA levels, the differences in drug resistance, or lack thereof (our study) may simply be due to cell type-specific differences, the oncogenic drivers or the triggers used. Investigating the role of PIDDosome components in other, preferentially, non-hematological tumor models will shed some light.

Prior work linked loss of Caspase-2 to chromosomal and genomic instability as a possible driver of accelerated transformation.^37^ Consistently, SV40 MEFs and c-Myc transgenic B cells lacking Caspase-2 showed significantly higher levels of micronuclei formation and numbers of chromosomes in metaphase spreads diverted more strongly from the normal number. However, our analysis of chromosome numbers in *Eμ-Myc* B-cell tumors did not show a direct increase in the frequency of aneuploidy in cells lacking *Caspase-2*. Instead, we found that the aneuploid cells present in the *Caspase-2-*deficient tumors showed a greater variation of chromosome numbers in the same tumor sample. Furthermore, our data show that the level of high-grade aneuploidy (i.e., a loss or gain of more than 10 chromosomes) was greater in *Caspase-2*^−*/*−^ tumors. Together, these results clearly support a possible role of Caspase-2 in maintaining genomic integrity, whereas loss of the adaptor protein Raidd is dispensable for genomic stability (not shown).

As it is difficult to connect these observations with known substrates of Caspase-2 and the analysis of representative p53-induced genes failed to support impaired murine double minute 2 activity, as reported in cisplatin-treated lung cancer cells,^40^ we performed whole transcriptome analysis on premalignant *Eμ-Myc* pre-B cells lacking or expressing Caspase-2 (or Raidd). However, we again failed to identify possible candidates that may be involved in the observed tumor-suppressor phenotype and established p53 targets were also not deregulated (Supplementary Figure 2). Hence, it remains possible that the phenomenon of reduced p53 activation in the absence of Caspase-2 is cell type-specific, for example, in lung epithelium,^40^ and/or a particular secondary hit, or a fully transformed cellular state.^37^ Consistent with the latter scenario, we previously reported on a reduced selective pressure to inactivate p53 in *Eμ-*Myc lymphomas lacking Caspase-2.^4^

In summary, our data suggest that Raidd does not influence the development of tumors after DNA-damage stress or the overexpression of oncogenes. This is in strong contrast to the opposing effects of its putative interaction partners, Caspase-2 or Pidd1.^3, 4^ Hence, the function of Raidd as a ‘direct activator of Caspase-2' needs to be re-considered, at least in the context of *Eμ-Myc-*driven tumorigenesis and justifies the search for additional activators as well as substrates of Caspase-2 to understand its tumor-suppressive function at the molecular level.

## Materials and Methods

### Mice

All animal experiments were performed in accordance to the Austrian legislation (BGBl. Nr. 501/1988 i.d.F. 162/2005, # BMWF-66.011/0137-II/10b/2009). The generation and genotyping of *Casp-2*^−/−^, *Raidd*^−/−^, *p53*^+/−^ and *Eμ-Myc* transgenic mice have been described elsewhere.^34, 44, 45, 46^ All mice used for the experiments were on an inbred C57BL/6 background.

### Tumorigenesis induced by DNA damage

To induce thymic lymphomas, mice at the age of 4 weeks irradiated with 1.75 Gy in a linear accelerator once per week for 4 weeks. Muscular sarcoma formation was induced by a single 200 *μ*l *intra muscular* injection of 1 mg 3-MC per mouse (Sigma, Vienna, Austria), dissolved in sesame oil. Mice treated with sesame oil alone (vehicle) served as a control.

### Cell culture and reagents

Primary tumor cells derived from *Eμ-Myc*, *Eμ-Myc*/*Raidd*^−*/*−^, *Eμ-Myc/Casp-2*^−*/*−^ and *Eμ-Myc/Pidd1*^−*/*−^ mice were cultured in DMEM (PAA, GE Healthcare, Pasching, Austria), supplemented with 10% FCS (PAA) Pen/Strep (Sigma), 250 *μ*M L-Glutamine (Gibco/Invitrogen, Vienna, Austria) and 50 *μ*M 2-mercaptoethanol (Sigma) and were kept on irradiated Bcl-2-overexpressing NIH-3T3 feeder cells. Tested agents and concentrations were: Etoposide (0.01–1 mg/ml), Dexamethasone (10^9^–10^7^ M), Paclitaxel (0.5–50 mM), Thapsigargin (0.5–50 ng/ml) and Doxorubicin (4–400 ng/ml; all from Sigma). FACS-sorted pre-B, immature and IgM^+^ B cells were cultured in DMEM with supplements and spontaneous cell death was monitored over time.

### Flow cytometric analysis and cell sorting

Tumors were analyzed by flow cytometry using the following cell surface markers for (a) B cells: RA3-6B2, anti-B220; R2/60, anti-CD43; II/41, anti-IgM; 11/26C, anti-IgD; MB19-1, anti-CD19; 53-7.3, anti-CD5 and B3B4, anti-CD23; 7E9, anti-CD21 (BioLegend, Fell, Germany); and (b) T cells: GK1.5, anti-CD4; H57-597, anti-TCRb (all from eBioscience, Vienna, Austria) and 53-6.7, anti-CD8; (from BD Pharmingen, San Diego, CA, USA). Biotinylated antibodies were monitored with streptavidin-RPE (DAKO, Vienna, Austria) or streptavidin-PE-Cy7 (BD Phamingen). Using a FACS^Vantage^ cell sorter (Becton Dickinson, Heidelberg, Germany) premalign pre/pro-B cells (B220^+^/IgM^−^) or immature (IgM^+^/IgD^low^) and mature (IgM^+^/IgD^high^) B cells were isolated and sorted from bone marrow and spleen, respectively. Flow cytometry data were analyzed using Cyflogic free ware and FlowJo (Ashland, OR, USA).

### Immunoblotting

Proteins were extracted from tumor cells for 1 h on ice in protein lysis-buffer.^36^ Insoluble debris was cleared by centrifugation for 5 min at 13 000 r.p.m. and 4 °C. For evaluation of the p53 status of tumor cells 30 *μ*g protein/lane was separated by SDS-PAGE, transferred to a nitrocellulose membrane and probed with rat anti-p19/ARF (5-C3-1; Santa Cruz Biotechnology, Szabo-Scandic, Vienna, Austria) or mouse anti-p53 antiserum (1C12; Cell Signaling, New England Biolabs, Frankfurt, Germany). Comparability of protein loading was assessed by re-probing membranes with an antibody recognizing GAPDH (Sigma). Horseradish peroxidase-conjugated sheep anti-rat Ig antibodies (Jackson Research, Vienna, Austria), goat anti-rabbit or rabbit anti-mouse antibodies (DAKO) were used as secondary antigens. Antibody binding was detected using enhanced chemiluminiscence (Amersham, Freiburg, Germany) system.

### Cell cycle analysis

Cell cycle analysis was based on fixing lymphoma cells in 70% ethanol and staining with PI (propidium iodide at 40 *μ*g/ml; Sigma). To analyze the percentage of cells in M-phase, ethanol-fixed lymphoma cells were permeabilized using Triton-X (0.25%, Sigma) for 15 min on ice and co-stained with phosphorylated H3 (Ser10; Cell Signaling) and PI. Distribution of cell cycle phases was analyzed using a FACScan cell cytometer (BD, Heidelberg, Germany).

### BrdU incorporation

Cell proliferation of immature (CD19^+^IgM^−^) and mature (CD19^+^IgM^+^) B cells in premalignant 4-week-old mice was assessed by injecting 1 mg BrdU/mouse *i.p*. Four hours later, primary cells derived from bone marrow and spleen were isolated and stained for BrdU incorporation using the BrdU/APC flow kit (BD, Vienna, Austria) according to the manufacturer's recommendation. Samples were analyzed using FACS-Calibur (BD).

### Cell viability assay

Tumor cells were co-stained with Annexin-V-FITC (1 : 1800 in Annexin-V-binding buffer; BD) and 7-AAD (1 *μ*g/ml, Sigma) and spontaneous cell death was analyzed by subsequent flow cytometric analysis.

### Cytogenetic analysis

Micronuclei were examined by seeding 2 × 10^5^ MEF on a sterile coverslip treated with cytochalasin (4 *μ*g/ml) for 16 h before fixation in PTEMF fixative^47^ for 10 min and staining with 4′,6′-diamidino-phenylinodole. A minimum of 300 cells per condition and genotype were screened for micronuclei under a ZEISS IMAGER Z1 Microscope and Zeiss EC Plan-NEOFLUAR 40 × /0,75 Ph2 objective using AxioVision Release 4.8.2 imaging software (Zeiss, Oberkochen, Germany).

Chromosome spreads from freshly isolated tumors were generated by incubating 1 × 10^6^ lymphoma cells in media containing 1 *μ*M nocodazole (Sigma) for 5 h at 37 °C in a CO_2_ incubator. After collecting and washing the cells in PBS, the pellet was resuspended in 5 ml 0.075 M KCl (Merck) and incubated for 5 min at 37 °C. Then 1 ml Carnoys fixative (Methanol:Acetic Acid=3:1) was added. Cells were spun down (1200 g for 3 min) and fixed in 1 ml Carnoys fixative (repeated three times). Cells were added drop-wise (from ∼50 cm hight) onto a coverslip, air dried and stained with 4′,6′-diamidino-phenylinodole. A minimum of 50 chromosome spreads per tumor sample was analyzed at × 100 magnification.

### qRT-PCR analysis

RNA was isolated using TRIzol (Invitrogen) and transcribed into cDNA (Omniscript, Qiagen, Hilden, Germany) using random hexamer primers after DNAse digestion (Promega, Mannheim, Germany). cDNA of interest was amplified using primers listed in Supplementary Table 1 and 2 × DyNAmo Color Flash SYBR Green Master Mix (Thermo Scientific, Vienna, Austria) in an Eppendorf Mastercycler ep realplex^2^ cycler. Relative expression of target/housekeeper was calculated using the delta-CT method.

### Microarray data set generation and analysis

Microarray data for gene expression were obtained using Affymetrix MoGene 1.0 ST v.1 arrays (Affymetrix, Santa Clara, CA, USA). Sample preparation was performed according to the manufacturer's protocol. In brief, 250 ng of high-quality RNA per sample were processed using the Ambion Affymetrix GeneChip WT Expression Kit (Part no. 308 4411974, Ambion/Thermo Scientific, Vienna, Austria) and the Affymetrix GeneChip WT Terminal Labeling Kit (Affymetrix). The resulting biotinylated targets were hybridized in an Affymetrix hybridization oven to a total of 14 Affymetrix MoGene 1.0 ST v.1 microarrays, which were then washed and stained in an Affymetrix fluidic station 450. Raw fluorescence signals were recorded in an Affymetrix scanner 3000 and image analysis was made with the Affymetrix GeneChip Command Console software.

Subsequent analyses have been performed in R (version 3.0.2) using packages from the Bioconductor project^48^ (version 2.13, BioConductor project: Fred Hutchinson Cancer Research Center, Seattle, WA, USA). The raw microarray data were pre-processed using ‘generalgcrma' package^49^ and our custom transcript-level ‘CEL definition file' (CDF) as described in Bindreither *et al.*^50^ Transcripts for all genes have been defined using Ensembl version 71 (EMBL-EBI, Wellcome Trust Genome Campus, Hinxton, UK). The CDF contained a total of 63 455 probe sets for 22 991 genes. Background adjustment, normalization and summarization of the microarray data were performed using the GCRMA method.^51^ Further, a single-probe set per gene was selected as following: (a) if there were probe sets with more than 5 probes, only those were considered for further analysis, (b) if any of considered probe sets represented protein coding transcripts, the list of probe sets was limited to such and (c) the probe set which had the highest multiple of average expression and standard deviation across all samples was chosen for expression analysis.

Differential expression analysis was performed using limma package.^52^ *P*-values were adjusted for multiple hypotheses testing correction by method of Benjamini and Hochberg.^53^ Genes with adjusted *P*-value<0.05 and absolute log2 fold change >1 were considered to be significantly regulated.

The raw and pre-processed microarray data have been submitted to the Gene Expression Omnibus (accession number GSE64920).

### Statistical analysis

Statistical analysis was performed using unpaired Student's *t*-test or ANOVA with Student–Newman–Keuls as *post hoc* test. Comparison of Kaplan–Meier survival plots was performed using a log-rank test and for evaluation of statistical difference in frequency distributions the *χ*^2^ (Fisher's exact) analysis algorithm was used. *P*-values of <0.05 (*) or *P*<0.001 (**) indicate significant differences.

## Abbreviations

PIDD: p53-induced protein with a death domain
RAIDD: receptor-interacting protein-associated ICH-1/CED-3 homologous protein with a death domain
NF-kB: nuclear factor kappa-light-chain enhancer
MEF: mouse embryonic fibroblast
wt: wild type
3-MC: 3-methylcholanthrene
