# Supplementary material for: The tumor-modulatory effects of Caspase-2 and Pidd1 do not require the scaffold protein Raidd
Source: Cell Death Differ. 2015 Apr 10;22(11):1803–11. doi: 10.1038/cdd.2015.31 (PMC4648327; doi:10.1038/cdd.2015.31)
Supplement: Supplementary Information [file cdd201531x1.pdf]

Supplemental Data:

Figure S1: Organ analysis of *Eµ-Myc* transgenic mice of different genotypes after tumor onset and evaluation of the functionality of the Myc/p19^{Arf}/p53 axis

Figure S2: Changes in transcription of p53 target genes upon loss of Caspase-2 or Raidd in c-Myc transgenic premalignant pre-B cells.

Figure S1:

(a) White blood cell count (WBC) in peripheral blood of diseased *Eµ-Myc* mice. Peripheral blood was stained with Turks solution (1:200) and counted in a hemocytometer. Data are given as WBC/µl. (b) Spleen weight in grams of mice with palpable *Eµ-Myc* tumors of the indicated genotypes. Numbers of animals analyzed are indicated. (c and d) Representative Western blot analysis of the tumor suppressor gene p53 and its target p19ARF in IgM^{+} and IgM^{-} tumor lysates derived from *Eµ-Myc* (c) and *Eµ-Myc/Raidd*$^{-/-}$ (d) mice. Reprobing with GAPDH was used as a control for loading. The alpha-numerical designations (*e.g.* H25) refer to the mouse ID number in our cohorts.

Figure S2:

(a) Relative mRNA levels of p21, Puma and Noxa in premalignant bone marrow-derived B220^{+}CD43^{+}IgM^{-} pre-B cells (n=3). (b) Vulcano blots of an Affymetrix Chip assay performed using mRNA from spleen-derived B220^{+}IgM^{+}IgD^{-} B cells isolated from indicated genotypes (n=3). Hits with adjusted p_{BH} – value <0.05 were considered as significantly changed. Adjustment was performed using Benjamini Hochberg method. Confirmative qRT-PCR analysis of hits identified in (b) using mRNA from B cells from *Eµ-Myc and Eµ-Myc/Casp-2*$^{-/-}$ (c) or *Eµ-Myc/Raidd*$^{-/-}$ (d) mice.

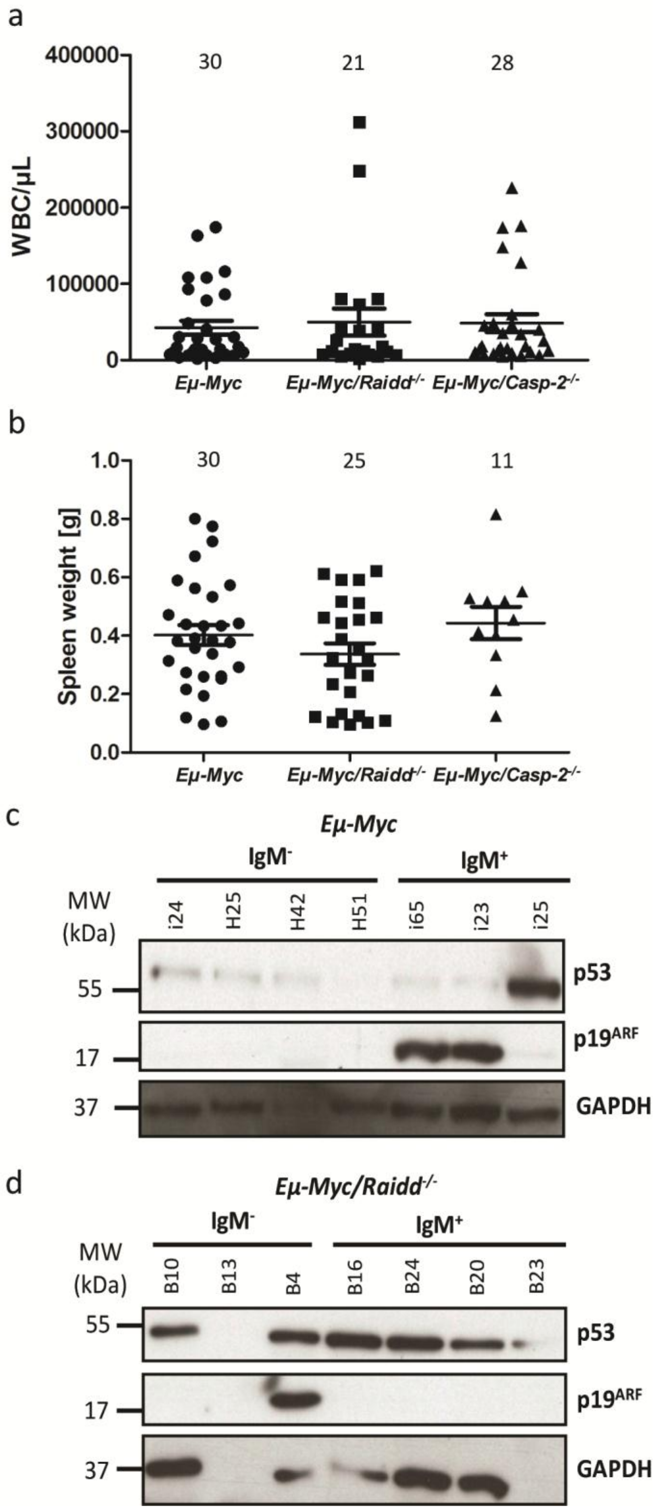
a
400000
300000
200000
100000
0
WBC/µL
30
21
28
Eµ-Myc
Eµ-Myc/Raidd-/-
Eµ-Myc/Casp-2-/-
b
1.0
0.8
0.6
0.4
0.2
0.0
Spleen weight [g]
30
25
11
Eµ-Myc
Eµ-Myc/Raidd-/-
Eµ-Myc/Casp-2-/-
c
Eµ-Myc
IgM-
IgM+
MW
(kDa)
i24
H25
H42
H51
i65
i23
i25
55
17
37
p53
p19ARF
GAPDH
d
Eµ-Myc/Raidd-/-
IgM-
IgM+
MW
(kDa)
B10
B13
B4
B16
B24
B20
B23
55
17
37
p53
p19ARF
GAPDH

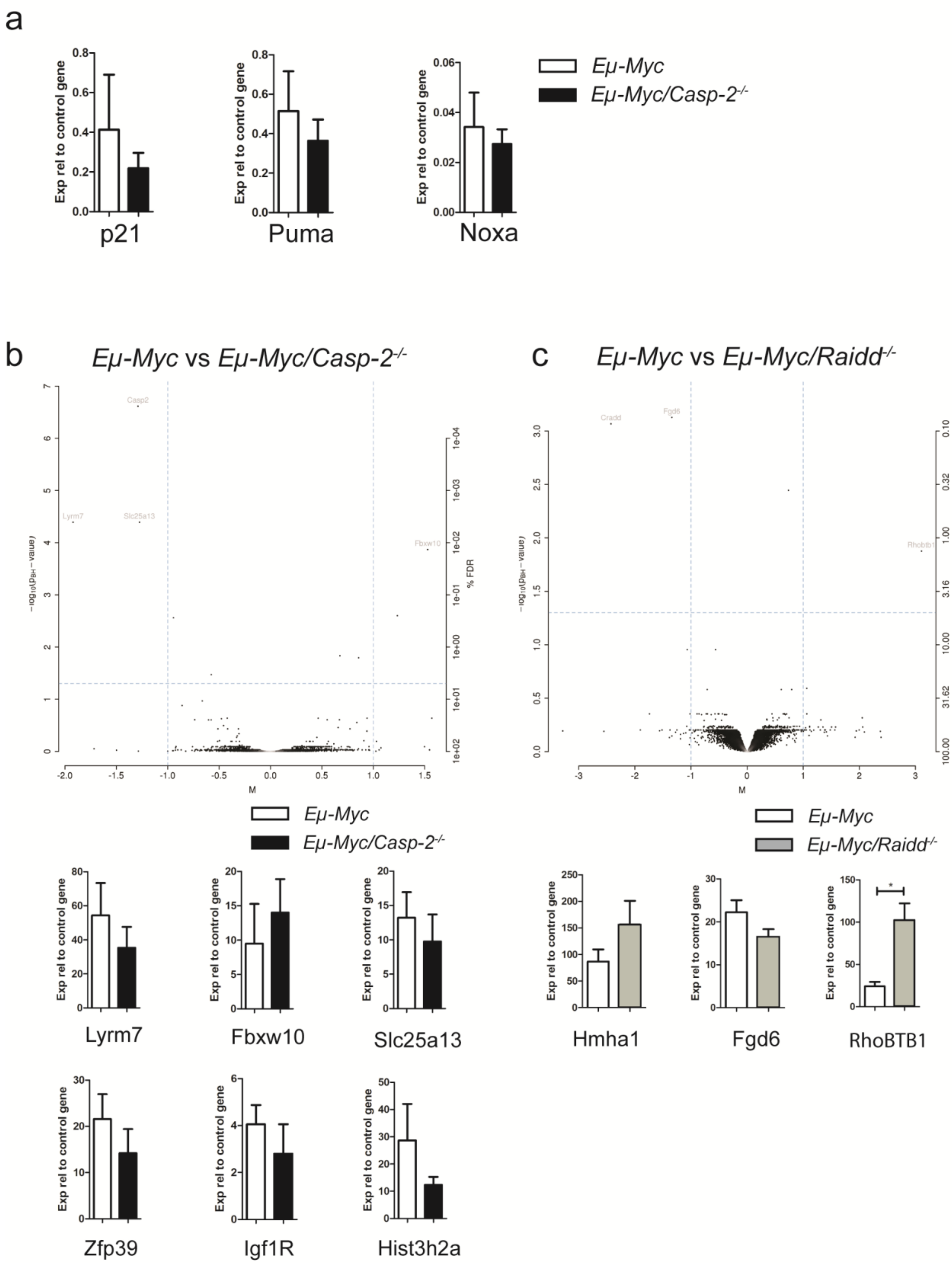
a
Exp rel to control gene
p21
Puma
Noxa
Eμ-Myc
Eμ-Myc/Casp-2-/-
b
Eμ-Myc vs Eμ-Myc/Casp-2-/-
Casp2
Lyrm7
Slc25a13
Fbxw10
M
% FDR
c
Eμ-Myc vs Eμ-Myc/Raidd-/-
Cradd
Fgd6
Rhobtb1
Eμ-Myc
Eμ-Myc/Casp-2-/-
Lyrm7
Fbxw10
Slc25a13
Zfp39
Igf1R
Hist3h2a
Eμ-Myc
Eμ-Myc/Raidd-/-
Hmha1
Fgd6
RhoBTB1

List of used Primers

Actin	fwd	5′- ACT GGG ACG ACA TGG AGAAG -3′	rev	5′- GGG GTG TTG AAG GTC TCA AA -3′
L32	fwd	5´- ATC CTG ATG CCC AAC ATC GG - 3´	rev	5´- CCA GCT GTG CTG CTC TTT CT - 3´
Fbxw10	fwd	5´- AAA GAC TCT CAC TGG GCA CG - 3´	rev	5´- AGC ACC TCC AGA GAC GAG AT - 3´
Fgd6	fwd	5´- TGT AAC CCG GAA GTG TCA GC - 3´	rev	5´- GCC TTT TTA AGC ACC CCT GC - 3´
Hist3h2a	fwd	5´- CGT GCT ACT GCC CAA GAA GA - 3´	rev	5´- AAA AGT CGC ACT AAC GAC CG - 3´
Hmha1	fwd	5´- CAG GAG TTG CAG GAC TGA GA - 3´	rev	5´- CTC GCT TCT TCC GAG AGA ACA - 3´
Igf1R	fwd	5´- GTG TGT GTC CTG GAT TTG GGA - 3´	rev	5´- GGC AGA AAT GCG GAG TGG AA - 3´
Lyrm7	fwd	5´- CAC GTT GCA AAT GAG CGA CA - 3´	rev	5´- AAG GGA AAG AGA GGA GCG TC - 3´
NOXA	fwd	5´- CCC ACT CCT GGG AAA GTA CA - 3´	rev	5´- AAT CCC TTC AGC CCT TGA TT - 3´
p21	fwd	5´- TTG CAC TCT GGT GTC TGA GC - 3´	rev	5´- TCT GCG CTT GGA GTG ATA GA - 3´
Puma	fwd	5´- CAA GAA GAG CAG CAT CGA CA - 3´	rev	5´- TAG TTG GGC TCC ATT TCT GG - 3´
RhoBTB1	fwd	5´- AAA GCC AAT GTC CTC CCT CG - 3´	rev	5´- ACG TTG GGT CGT TCG TAG TC - 3´
Slc25a13	fwd	5´- AGC CCA GAA GTT TGG TCA GG - 3´	rev	5´- CTC GGC CAA GTT GAA GGG TA - 3´
Zfp39	fwd	5´- CTG GGT ACG GGC AGA AAC TT - 3´	rev	5´- CGA CTC CTG CAT GTA CCC TC - 3´
